# Optogenetics design of mechanistically-based stimulation patterns for cardiac defibrillation

**DOI:** 10.1038/srep35628

**Published:** 2016-10-17

**Authors:** Claudia Crocini, Cecilia Ferrantini, Raffaele Coppini, Marina Scardigli, Ping Yan, Leslie M. Loew, Godfrey Smith, Elisabetta Cerbai, Corrado Poggesi, Francesco S. Pavone, Leonardo Sacconi

**Affiliations:** 1 European Laboratory for Non-Linear Spectroscopy, 50019 Sesto Fiorentino, Italy; 2 National Institute of Optics, National Research Council, 50125 Florence, Italy; 3 Division of Physiology, Department of Experimental and Clinical Medicine, University of Florence, 50134 Florence, Italy; 4 Division of Pharmacology, Department “NeuroFarBa,” University of Florence, 50139 Florence, Italy; 5 R. D. Berlin Center for Cell Analysis and Modeling, University of Connecticut Health Center, Farmington, CT 06030, USA; 6 Institute of Cardiovascular and Medical Sciences, University of Glasgow, Glasgow, UK; 7 Department of Physics and Astronomy, University of Florence, 50019 Sesto Fiorentino, Italy

## Abstract

Current rescue therapies for life-threatening arrhythmias ignore the pathological electro-anatomical substrate and base their efficacy on a generalized electrical discharge. Here, we developed an all-optical platform to examine less invasive defibrillation strategies. An ultrafast wide-field macroscope was developed to optically map action potential propagation with a red-shifted voltage sensitive dye in whole mouse hearts. The macroscope was implemented with a random-access scanning head capable of drawing arbitrarily-chosen stimulation patterns with sub-millisecond temporal resolution allowing precise epicardial activation of Channelrhodopsin2 (ChR2). We employed this optical system in the setting of ventricular tachycardia to optimize mechanistic, multi-barrier cardioversion/defibrillation patterns. Multiple regions of conduction block were created with a very high cardioversion efficiency but with lower energy requirements as compared to whole ventricle interventions to interrupt arrhythmias. This work demonstrates that defibrillation energies can be substantially reduced by applying discrete stimulation patterns and promotes the progress of current anti-arrhythmic strategies.

Life-threatening arrhythmias, including atrial and ventricular fibrillation, represent a social and economic burden in the general population. The electro-anatomical substrate of these arrhythmias is characterized by conduction heterogeneities that facilitate micro and macro re-entrant circuits^1^. Electrical cardioversion, attempted either with external or implantable devices, is the first choice option according to current guidelines^2^. However, electrical cardioversion operates ignoring the characteristic features of re-entrant conduction pathways and its efficacy rests on capturing the whole heart with a generalized electrical discharge^3^. For instance, implantable cardioverter defibrillator (ICD) shocks cause intense pain, myocardium damage, and chest muscle contractures, with a non-negligible risk of accidents and often unbearable psychological discomfort^4^; moreover, inappropriate ICD-shocks are regrettably common^5^. In this scenario, efforts towards minimizing ICD’s electrical entrainments require a deeper comprehension of the events leading to the onset and development of arrhythmias. Light provides the versatility required to develop and test customized stimulation patterns capable of successfully interrupting arrhythmias at much lower energy. We exploit recent advancements in optogenetics^6^,^7^,^8^ and imaging techniques^9^,^10^ to develop a novel optical platform capable of simultaneously mapping and controlling the electrical activity of whole hearts with sub-millisecond temporal resolution. An ultra-fast laser scanning system is used to design arbitrarily-chosen stimulation patterns across the whole heart. The platform is then used to characterize arrhythmias and to efficiently restore the cardiac sinus rhythm by intervening with customized stimulation patterns.

## Results

**Figure 1:**
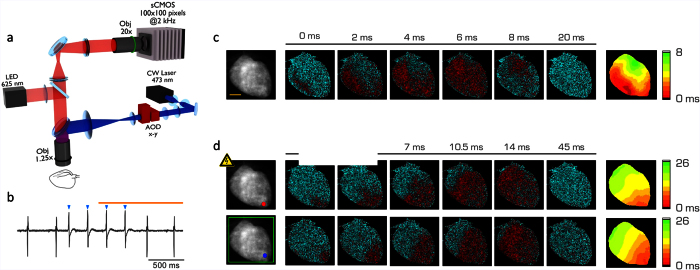
Simultaneous all-optical map and control of cardiac conduction pathway. (**a**) Scheme of the wide-field fluorescence macroscope consisting of a LED at a wavelength centered at 625 nm for excitation of the voltage-sensitive dye and a CW laser at 473 nm for ChR2 activation. The system is provided with a random access scanning head comprised of two orthogonally mounted acousto-optical deflectors (AOD x-y). A 1.25x objective is used for imaging and stimulation of the whole mouse heart, a 20x objective is used to focus the fluorescence signal into a central portion (100 × 100 pixel) of a sCMOS sensor operating at a frame rate of 2 kHz. (**b**) Electrocardiographic recording of a ChR2-expressing heart, showing sinus rhythm with a frequency of 3 Hz. Blue arrowheads represent the ChR2 stimulation performed at 5 Hz (laser power at the sample 4.9 mW, illumination time 5 ms). The orange line represents the LED illumination. (**c**) Fluorescence image (F_0_) of a ChR2-expressing heart stained with voltage sensitive dye (left). Scale bar of 2 mm in yellow. Six representative frames of optical mapping (ΔF/F_0_) recorded from the same heart. The electrical activation is reported in red and the baseline in cyan. On the right, corresponding color-scaled isochronal map of the action potential reporting the voltage activation time per pixel. (**d**) On the left, fluorescence image (F_0_) of a ChR2-expressing heart stimulated in the apex either with an electrode (red spot, above) or with ChR2 activation with the blue laser (blue spot, below). The green frame represents the AODs working-field on the sample. Corresponding optical mapping frames (ΔF/F_0_) recorded from the same heart are reported next. On the right, corresponding color-scaled isochronal map of the action potential reporting the activation time per pixel.

A wide-field macroscope (Fig. 1a) operating at 2,000 frames per second was developed and used to map the action potential propagation in Langendorff’s horizontally-perfused mouse hearts loaded with a red-shifted voltage sensitive dye (di-4-ANBDQPQ)^11^. Sinus rhythm in perfused hearts was characterized by regular and rapid activation of the ventricles monitored by electrocardiographic (ECG) recordings (Fig. 1b, Figure S1, Table S1, Video S1) and propagation maps (Fig. 1c). To optically control the cardiac electrical activity, we generated transgenic mice expressing the photo-sensitive ion channel, Channelrhodopsin 2 (ChR2)^12^ and exploited light-induced depolarization as one means for localized electrical discharges. We first compared the activation features of a point source stimulation, induced either with an electrode or with ChR2 photo-activation. Electrical and optical point stimulation evoked similar isochronal maps (Fig. 1d, Videos S2–3), suggesting that the ChR2 activation represents an effective alternative to the electrical stimulation. Notably, the use of the red-shifted voltage sensitive dye ensured that its excitation wavelength (640 nm) was unable to generate unwanted ChR2 activation (Fig. 1b, Table S1).

**Figure 2:**
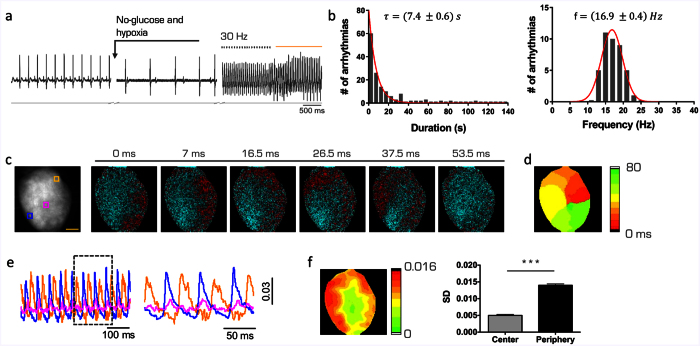
Induction and characterization of Ventricular Tachycardia (VT). (**a**) Representative electrocardiographic recording of a mouse heart. Sinus rhythm shows a frequency of 2.7 Hz and upon change of perfusion to the hypoxic and glucose-deprived solution, the heart shows a partial atrio-ventricular block with a non-regular activation and a mean frequency of 1.85 Hz. An electrode is used to provide a burst of high frequency stimulation (20 pulses at 30 Hz, black arrows). The ECG shows the onset of a sustained VT. The orange line represents the LED illumination. (**b**) Distribution of duration (left) and frequency (right) of VTs. Data from 149 VT in 16 hearts (graph of duration) and from 64 VTs in 14 hearts (frequency). The red lines represent the best fit of the distribution performed with a mono-exponential decay for durations and with a Gaussian function for frequencies. The best τ and mean frequency are reported in the figure ± SEM (standard error of the mean). (**c**) Optical mapping during VT. (**d**) Corresponding color-scaled isochronal map. (**e**) Fluorescence signal (ΔF/F_0_) of the three regions of interest reported in (**c**). Each region corresponds to 5 × 5 pixel equal to 0.25 mm^2^. A close-up of the signals (interval indicated by the dashed box). (**f**) Pixel-per-pixel map of the amplitude signal quantified using standard deviation of the mean (SD). Graph showing an average of signal SD detected in the center and along the pathway of the re-entrant circuit. Data reported as mean ± SEM from 6 hearts. Student’s T-test applied (***p < 0.001).

We then experimentally induced arrhythmias by rapid ventricular pacing during perfusion with a glucose- and oxygen-free solution, mimicking ischemic conditions^13^. This solution induced alterations of the electrical propagation in the form of partial atrio-ventricular block and ventricular ectopies or short-runs of non-sustained ventricular tachycardia (VT) (Fig. 2a). Then, a short stimulation burst at 20–40 Hz in the left ventricular (LV) epicardium was provided to trigger sustained VT (Fig. 2a,b). This strategy generated monomorphic sustained VTs with a duration distribution characterized by a mono-exponential decay (τ = 7.4 ± 0.6 *s*), and a frequency distribution centered at 16.9 ± 0.4 Hz. As expected^14^, optical mapping recordings revealed a single large re-entrant circuit that occurred either clockwise or counterclockwise (Fig. 2c, Videos S4–5). In fact, consistent with the conduction velocity (~0.5 m/s) and refractory period of action potentials (~50 ms) in mouse hearts^15^, a reentry circuit could self-sustain only invading the entire LV free wall. The action potential propagation during the re-entrant conduction was much slower than in normal sinus rhythm (Fig. 2d). The central region of the re-entrant circuit showed no electrical activity (Fig. 2e,f and Figure S2) due to the spiral wave core properties.

Inspired by surgical approaches that interrupt re-entrant circuits with linear scars perpendicular to the excitation wavefront (i.e. maze procedures^16^), we augmented the optical macroscope with a laser scanning system based on acousto-optics deflectors^9^,^10^,^17^ (AODs, Fig. 1a) to draw ChR2 conduction-block lines on the arrhythmic heart. The idea was to exploit the transient refractoriness produced by the ChR2 stimulation to interrupt the approaching re-entry wavefront. The maximum capture rate of ChR2 stimulation in myocardium is 5–15 Hz^8^,^12^ (see Figure S3). Thus, a 10 Hz ChR2 stimulation was applied with a triple-line pattern to provide multiple block fronts in order to increase the probability that the re-entry wavefront collided with a refractory territory. Since the goal of our study was to provide new insights for improving the electrical defibrillation technology based on selective stimulation patterns, we decided to exclude hyperpolarizing opsin^18^. The *triple-barrier* was compared with a *single point* ChR2 stimulation in the center of the re-entrant circuit, as previously achieved with contact electrodes^19^ or optogenetic stimulation^20^, and with a *whole LV* activation, similar to the one achieved with electrical cardioversion shocks^3^,^21^.

**Figure 3:**
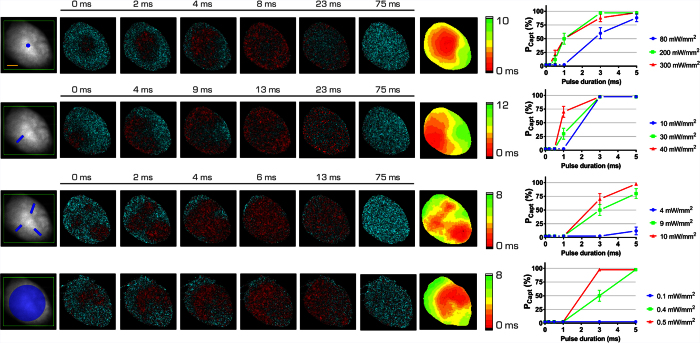
Optical manipulation of the cardiac conduction pathway. Four patterns of optogenetic stimulation: *single-point*; *single-barrier*, *triple-barrier*, and *whole LV*, designed as reported by the blue traits on the fluorescence image (F_0_) of the heart. The green frame represents the AODs working-field on the sample. Corresponding frames of fluorescence signal (ΔF/F_0_) are reported next with the relative isochronal maps. For each isochronal map the scale has been individually adjusted to better highlight the activation times of every pattern. On the right, capture probability (P_capt_) for each ChR2 stimulation pattern based on pulse duration and laser power. P_capt_ is defined as the percentage of all flashes given that successfully captured the ventricle. Different colors (blue, green, and red) depict different laser intensities. Note that the laser power is maintained constant for each color for every pattern (blue = 1.3 mW, green = 3.4 mW, and red = 4.9 mW), with the exception of the *whole LV* illumination, in which laser power has been increased four times. Data reported as mean ± SEM (standard error of the mean) from N = 3. Scale bar of 2 mm in yellow.

Before applying the ChR2 stimulation for interrupting VT, we characterized the response of the heart to different ChR2 patterns in terms of illumination intensity, pulse duration (dose-response curves), as well as the evoked alteration in propagation maps (Fig. 3, Videos S6–9). We found that only the *triple-barrier* and the *whole LV* illumination protocols effectively generated activation times comparable to sinus rhythm while the *single point* and *single line* evoked activation maps displayed significantly delayed activation times. To prove that no effects are caused by the illumination itself, the same stimulation patterns have been also applied in hearts not expressing ChR2 and stained with the voltage-sensitive dye. Even though the highest energy-dose settings reported in Fig. 3 was used, no alteration of the electrical activity has been observed in the ECG (number of hearts = 6).

**Figure 4:**
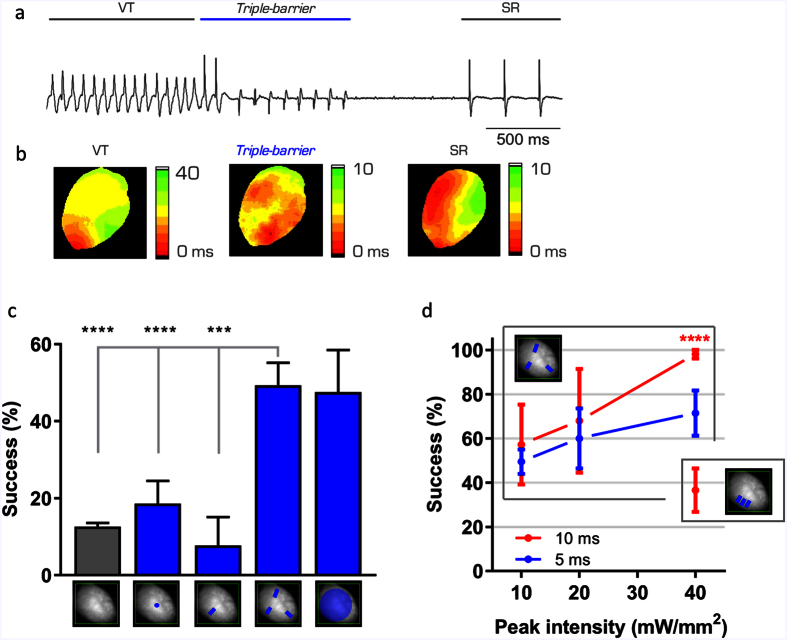
*Customized* pattern design for VT interruption. (**a**) Example of ECG recording showing a cardioversion. After the onset of the VT, the *triple-barrier* pattern was applied for 1 s at 10 Hz. The heart stops showing VT and restores its own sinus rhythm. (**b**) Isochronal maps relative to the ECG recording reported in (**a**). Isochrones depicting the VT (left), the ChR2 stimulation (middle), and the restored sinus rhythm (right). (**c**) Graph showing the percentage of spontaneous cardioversion of VTs (gray bar) and VTs interrupted with each of the four patterns reported in the x-axis (blue bars). Data reported as mean ± SEM (standard error of the mean) from: n = 43 and N = 5 (*single-point*); n = 13 N = 3 (*single-barrier*); n = 71 N = 4 (*triple-barrier*), n = 21 N = 3 (*whole LV*). 1-way ANOVA test applied (***p < 0.001, ****p < 0.0001). (**d**) Graph showing the success of cardioversion of the *triple-barrier* using different laser intensities and two different pulse duration: 5 ms (blue) and 10 ms (red). Success of cardioversion using 40 mW/mm^2^ of laser intensity and 10 ms of pulse duration has also been tested positioning the three lines parallel and close to each other as reported in the inset. Student’s T-test has been applied for comparison (****p < 0.0001 in red).

ChR2 stimulation was then applied in the setting of induced VT. Based on the distribution of VT durations (Fig. 2b), we awaited 5 s after the VT onset before applying the cardioversion protocols. We used a burst of ten pulses (5 ms of illumination time per pulse) at 10 Hz (1 s of total intervention) adjusting the laser power for every ChR2 stimulation pattern in order to assure a 100% capture success (Fig. 3). This choice of settings introduced a uniform threshold for all the different ChR2 stimulation patterns, allowing us to perform a comparative study. We found that the *triple-barrier* effectively interrupted VT restoring sinus rhythm in approximately 50% of the cases, while *single point* and *single barrier* configurations produced a cardioversion success rate that was not statistically different from the spontaneous one (Fig. 4). The *triple-barrier* stimulation (applying a laser intensity of 10 mW/mm^2^) showed a cardioversion success rate comparable to that achieved with *whole LV* illumination (stimulated at 0.5 mW/mm^2^), but with a significant confinement of the irradiated epicardium (Figure S4) and reduced total irradiation energy (0.25 *mJ* and 1 *mJ*, respectively). These findings highlight the importance of customized stimulation patterns based on arrhythmogenic mechanisms to lower stimulation energy. Once the best stimulation pattern was defined (for this single-reentry case), the cardioversion efficiency was optimized. The *triple-barrier* stimulation achieves a cardioversion success of (98.1 ± 2.0) % with a 4-fold increase of the light power and a 2-fold increase of the illumination time per pulse (Fig. 4d) which corresponded to a total irradiation energy of 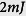. For comparison, using a protocol in which the heart is entirely illuminated (~1 cm^2^) with continuous illumination (~1 s) at low intensity (~0.5 mW/mm^2^), the total irradiation energy would be 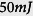. To further confirm that the cardioversion efficiency was stringently dependent on the mechanistic-based design, we positioned three lines regardless of the re-entry wavefront using the same total irradiation energy, obtaining a cardioversion rate of only (36.6 ± 9.8) %.

## Discussion

Although optogenetics represents a useful tool for basic research, there are multiple barriers to using cardiac ChR2 expression by viral gene transfer^22^ in humans soon. Here, we describe a method using optogenetics in combination with a red-shifted voltage sensitive dye to simultaneously map and control cardiac electrical activity with a high temporal resolution. Additionally, the two orthogonal AODs give us the freedom to rapidly move the laser to design any desired ChR2 stimulation pattern. Our optical toolkit establishes an innovative approach that overcomes limitations present in previous studies^23^,^24^. Nussinovitch and Gepstein^8^ employed optogenetics for cardiac pacing and resynchronization, simultaneously monitoring action potential propagation in whole mouse hearts. However, their work lacks the spatial control of ChR2 stimulation and means to designing patterns. Alternatively, the inspiring work by Burton *et al.*^7^ took advantage of digital micromirror device to manipulate light positioning to control electrical waves in a real simultaneous manner. However, they tested their optical platform on cardiac monolayer cultures with slower spiral waves. The system described in this study allows for full optical control at sub-ms temporal resolution to assess and investigate cardiac arrhythmias in whole isolated mouse hearts.

We induced arrhythmias mimicking ischemic conditions by reducing oxygen and depriving the heart of glucose. Ischemia affects ionic currents and concentrations, leading to local heterogeneity in excitability and action potential conduction^25^. We observed functional reentry circuits generating monomorphic VT, lasting from a few seconds to a few minutes. The regularity and repeatability of VTs allowed us to characterize the reentry circuits established in each heart. To date, electrical resetting of the heart has been the rule to successfully treat VTs of any cause. In the present work, we prove the potential of mechanistically-based electrical discharges to treat VTs, while assuring the same efficacy as whole heart cardioversion. By means of light-induced depolarizations, we demonstrate that VTs can be interrupted reducing the irradiated area and the total irradiation energy. We show that for simpler monomorphic VT circuits, rational protocols can be designed and optimized to achieve a very high cardioversion success. Mechanistic knowledge about the underlying arrhythmia helps reduce energy, and the cardioversion success dramatically drops if the pattern is designed regardless of the shape of the re-entrant circuit, even though the same amount of irradiated area and irradiation energy are employed.

Since cardiac electrophysiology in the mouse is substantially different from that in larger mammals, including humans, we provide an all-optical platform that can be scaled to larger hearts. The proposed concept, possibly implemented with red-shifted ChR2 (with higher tissue penetration depth)^26^, could be also applied in larger hearts, where more complex stimulation patterns may be required to address more complex arrhythmias such as ventricular fibrillation. Larger mammals could also open new experimental routes for the study of atrial arrhythmias, where line placement (e.g. around pulmonary veins^27^) is crucial. Optogenetic defibrillation has been envisioned since the attainment of ChR2 expression in the heart and predicted *in silico*^28^. We provide the first demonstration that optogenetics in the heart can be employed to investigate methods for efficiently interrupting arrhythmias and, more importantly, we prove that defibrillation energies can be reduced when reentry-based interventions are applied. We propose an enabling technology for seeking novel defibrillating strategies and further current anti-arrhythmic technology.

## Methods

### Mouse Model generation

Transgenic adult mice (3 months old) with a genetic background C57B6J and expressing cre-recombinase under the control of the α-MyHC promoter were bred with B6.Cg-Gt(ROSA)26Sortm27.1(CAG-COP4*H134R/tdTomato)Hze/J expressing mice (Jackson Lab). The resulting offspring had the STOP cassette deleted in their heart, resulting in cardiac expression (α-MyHC-promotor driven) of the hChR2(H134R)-tdTomato fusion protein. All animal procedures performed conformed with the guidelines from the Directive 2010/63/EU of the European Parliament on the protection of animals used for scientific purposes; the experimental protocol was approved by the Italian Ministry of Health (approved protocol number 647/2015-PR).

### Isolated and perfused mouse heart

Transgenic positive and negative mice (8 weeks old) were heparinized (0.1 mL at 5,000 units/mL) and anesthetized by inhaled isoflurane (5%). The excised heart was immediately bathed in Krebs-Henseleit (KH) solution and cannulated through the aorta. The KH buffer contained (in mM): 120 NaCl, 5 KCI, 2 Mg_2_SO_4_-7H_2_O, 20 NaHCO_3_, 1.2 NaH_2_PO_4_-H_2_O, and 10 glucose pH 7.4 when equilibrated with carbogen (95% O_2_–5% CO_2_). The contraction was inhibited for the entire experiment with blebbistatin (5 μM) in the solution. The cannulated heart was retrogradely perfused (Langendorff’s perfusion) with the KH solution and then transferred to a custom-built optical mapping chamber at a constant flow of 1.2 mL/min at 37 °C. Two platinum electrodes were placed below the heart for monitoring cardiac electrical activity via electrocardiogram (ECG). After stabilization of the electrocardiogram, typically within a few seconds, 1 ml of perfusion solution containing the voltage sensitive dye (di-4-ANBDQPQ; 50 μg/ml) was bolus injected into the aorta.

### Ventricular Tachycardia (VT) induction and ECG characterization

To induce ventricular tachycardia, we replaced KH perfusion with a Tyrode solution deprived of glucose and without carbogen aeration. The Tyrode solution contained (in mM): 113 NaCl, 4.7 KCl, 1.2 MgCl_2_, and 10 HEPES supplemented with 5 μM blebbistatin; pH adjusted to 7.35 with NaOH. Moreover, a high frequency (30–40 Hz) stimulation burst was applied by using a bipolar tungsten electrode placed on the surface of the right ventricle. VT duration and frequency were assessed using the ECG. Spontaneous cardioversion reported in Fig. 4c was estimated by determining the probability of VT interruption within one second (duration of every cardioversion protocol) based on the mono-exponential decay of the duration distribution.

### All-optical imaging and manipulation platform

Optical mapping and manipulation were performed using a custom-made upright macroscope (Fig. 1a). The whole mouse heart was excited in wide-field configuration using a 1.25x objective (EC Plan-Neofluar 1.25x/0.03 M27, Carl Zeiss Microscopy) and a LED operating at a wavelength centered at 625 nm (M625L3, Thorlabs) followed by a band-pass filter at 640/40 nm (FF01-640/40-25, Semrock). A dichroic beam splitter (FF685-Di02-25 × 36, Semrock) followed by a band-pass filter at 775/140 nm (FF01-775/140-25, Semrock) were used for collecting the emitted fluorescence. A 20x objective (LD Plan-Neofluar 20x/0.4 M27, Carl Zeiss Microscopy) was used to focus the fluorescence in a central portion (100 × 100 pixel) of a sensor of a sCMOS camera (OrcaFLASH 4.0, Hamamatsu Photonics) operating at frame rate of 2 kHz (506 μs actual exposure time). To manipulate electrical activity, a 473 nm CW laser (OBIS 473 nm LX 75 mW, Coherent) for ChR2 activation was overlaid to the imaging path using a second dichroic beam splitter (FF484-FDi01-25 × 36, Semrock). The laser beam waist after the objective lens was ≈100 μm diameter. The system was provided with a random access scanning head, comprised of two orthogonally mounted acousto-optical deflectors (AODs, DTSXY400, AA Opto-Electronic). For triple-barrier stimulation patterns, the AODs rapidly scanned lines with a commutation time ≈5 μs between a line and the next. The integration time on each line was 0.1 ms. After scanning the first three lines, the AODs returned to their initial position and repeated the cycle for the total illumination time. For whole LV illumination, the first lens after the AODs was removed and the laser beam was focused in the back focal plane of the objective lens in order to provide a homogeneous illumination with a diameter ≈6.5 mm. A custom-made computer software (LabVIEW 2014, National Instruments) was used for controlling the apparatus.

### Image processing

ΔF/F_0_ imaging of the electrical activity in the heart was performed processing the 8-bit depth raw stack with an ImageJ plugin (IO and VSD signal processor) originally developed for brain activity imaging^29^. During this processing, no temporal and spatial averaging was performed. Each fame was normalized to the mean baseline yielding a video a percent changes in fluorescence over time. Isochronal maps were performed using a custom-made software written in LabVIEW 2014. First, the software performed a spatial averaging using a Gaussian filter with a kernel size of (7 × 7) pixels. Then, a seed reference pixel was arbitrarily chosen and, pixel by pixel, the cross-correlation of the fluorescence trace was calculated in order to estimate the temporal shift among every pixel.

## Additional Information

**How to cite this article**: Crocini, C. *et al.* Optogenetics design of mechanistically-based stimulation patterns for cardiac defibrillation. *Sci. Rep.* **6**, 35628; doi: 10.1038/srep35628 (2016).

## Supplementary Material

Supplementary Information

Supplementary Video 1

Supplementary Video 2

Supplementary Video 3

Supplementary Video 4

Supplementary Video 5

Supplementary Video 6

Supplementary Video 7

Supplementary Video 8

Supplementary Video 9
