# Supplementary material for: Optogenetics design of mechanistically-based stimulation patterns for cardiac defibrillation
Source: Sci Rep. 2016 Oct 17;6:35628. doi: 10.1038/srep35628 (PMC5066272; doi:10.1038/srep35628)
Supplement: Supplementary Information [file srep35628-s1.pdf]

Optogenetics design of mechanistically-based stimulation patterns for cardiac defibrillation

Claudia Crocini, Cecilia Ferrantini, Raffaele Coppini, Marina Scardigli, Ping Yan, Leslie M. Loew, Godfrey Smith, Elisabetta Cerbai, Corrado Poggesi, Francesco S. Pavone, and Leonardo Sacconi

Supplementary figures

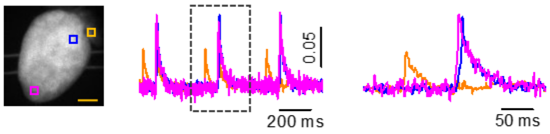

Figure S1: Optical mapping and Signal-to-Noise ratio in action potential detection. Fluorescence image (F_0) of a mouse heart stained with the voltage sensitive dye (left). Scale bar of 2 mm in yellow. Fluorescence signal ($\Delta F/F_0$) corresponding to the three regions of interest reported in the image on the left: apex (magenta), base (blue) and atrium (yellow). Each region corresponds to 250 μm^2. A close-up of the signals (interval indicated by the dashed box) is reported on the right.

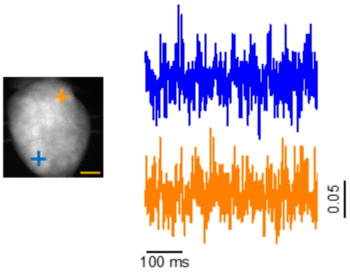

Figure S2: Signal-to-Noise ratio at single pixel resolution. Fluorescence image (F_0) of a mouse heart stained with the voltage sensitive dye (left). Scale bar of 2 mm in yellow. Fluorescence signal ($\Delta F/F_0$) recorded during VT corresponding to one single pixel from the two regions reported in the image on the left.

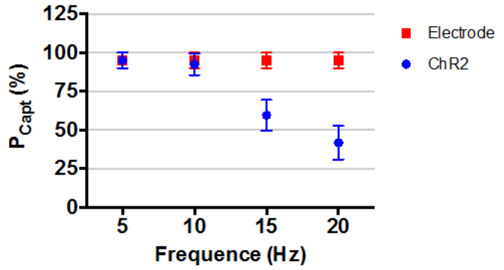

Figure S3: Maximum stimulation frequency with optogenetics. Optogenetics capture probability (P_{capt}) of heart stimulation based on ChR2 (blue) and electrode (red). Stimulation duration of 3 ms for both ChR2 and electrode. ChR2 stimulation was performed using *single-point* stimulation at 300 mW/mm^2. Data reported as mean ± SEM (standard error of the mean) from N = 3.

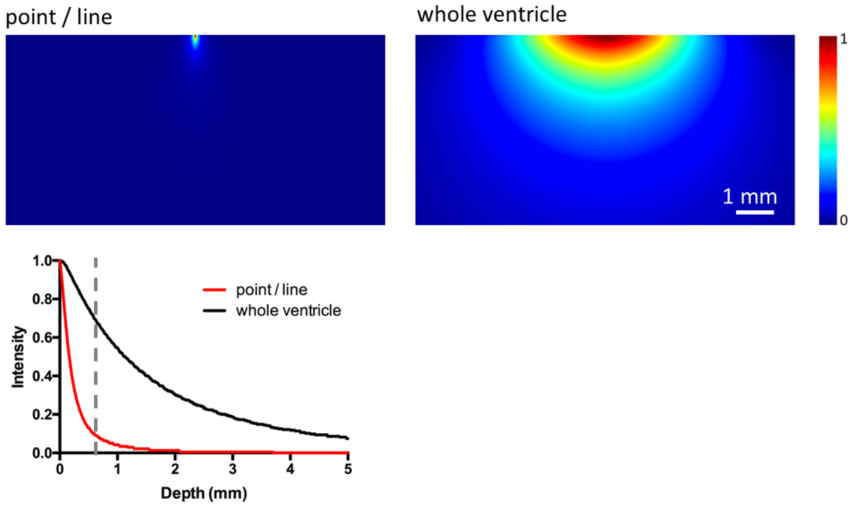

Figure S4: Simulation of the blue light propagation through the epicardium. Simulation of the irradiance of the 473 nm CW laser into the myocardium with point/line or whole ventricle designs obtained with Virtual Tissue Simulator that is based on Monte Carlo simulation (VTS; http://www.virtualphotonics.org/). The graph shows the normalized intensity vs. depth at central position of the illumination beam. Grey dashed line represents the left ventricular wall thickness in 8-weeks mice hearts. In this simulation a reduced scattering coefficient (μ_s') of 14.8 mm^{-1} and refractive index of 1.4 was used.

Supplementary videos

Video S1: Sequence of 150 fluorescence images (F_0) recorded at 2 kHz of a ChR2-expressing heart stained with the voltage sensitive dye. The electrical activation is reported in red and the baseline in cyan and is generated by the heart sinus rhythm. Six selected frames of this video are reported in figure 1c.

Video S2: Sequence of 150 fluorescence images (F_0) recorded at 2 kHz of a ChR2-expressing heart stained with the voltage sensitive dye. The electrical activation is reported in red and the baseline in cyan and is generated by an electrode placed at the apex. Six selected frames of this video are reported in figure 1d (upper panel).

Video S3: Sequence of 150 fluorescence images (F_0) recorded at 2 kHz of a ChR2-expressing heart stained with the voltage sensitive dye. The electrical activation is reported in red and the baseline in cyan and is generated by ChR2 activation with the blue laser in the apex. Six selected frames of this video are reported in figure 1d (panel below).

Video S4: Sequence of 120 fluorescence images (F_0) recorded at 2 kHz of a mouse heart stained with the voltage sensitive dye. The electrical activation is reported in red and the baseline in cyan and is recorded during a clockwise functional reentry circuit.

Video S5: Sequence of 120 fluorescence images (F_0) recorded at 2 kHz of a mouse heart stained with the voltage sensitive dye. The electrical activation is reported in red and the baseline in cyan and is recorded during a counterclockwise functional reentry circuit. Six selected frames of this video are reported in figure 2c.

Video S6: Sequence of 150 fluorescence images (F_0) recorded at 2 kHz of a ChR2-expressing heart stained with the voltage sensitive dye. The electrical activation is reported in red and the baseline

in cyan. The electrical activation is generated by ChR2 stimulation performed with the blue laser in the centre of the heart. Six selected frames of this video are reported in figure 3.

Video S7: Sequence of 150 fluorescence images (F_0) recorded at 2 kHz of a ChR2-expressing heart stained with the voltage sensitive dye. The electrical activation is reported in red and the baseline in cyan. The electrical activation is generated by ChR2 stimulation with blue laser in the shape of a single line. Six selected frames of this video are reported in figure 3.

Video S8: Sequence of 150 fluorescence images (F_0) recorded at 2 kHz of a ChR2-expressing heart stained with the voltage sensitive dye. The electrical activation is reported in red and the baseline in cyan. The electrical activation is generated by ChR2 stimulation with blue laser in the shape of three lines. Six selected frames of this video are reported in figure 3.

Video S9: Sequence of 150 fluorescence images (F_0) recorded at 2 kHz of a ChR2-expressing heart stained with the voltage sensitive dye. The electrical activation is reported in red and the baseline in cyan. The electrical activation is generated by ChR2 stimulation with blue laser in the shape of a large spot illuminating the whole left ventricle. Six selected frames of this video are reported in figure 3.
